# Self-Organized Discrimination of Resources

**DOI:** 10.1371/journal.pone.0019888

**Published:** 2011-05-18

**Authors:** Alexandre Campo, Simon Garnier, Olivier Dédriche, Mouhcine Zekkri, Marco Dorigo

**Affiliations:** 1 IRIDIA-CoDE (Institut de Recherches Interdisciplinaires et de Développements en Intelligence Artificielle, Department of Computer and Decision Engineering), Université Libre de Bruxelles, Brussels, Belgium; 2 Department of Ecology and Evolutionary Biology, Princeton University, Princeton, New Jersey, United States of America; University of Sheffield, United Kingdom

## Abstract

When selecting a resource to exploit, an insect colony must take into account at least two constraints: the resource must be abundant enough to sustain the whole group, but not too large to limit exploitation costs, and risks of conflicts with other colonies. Following recent results on cockroaches and ants, we introduce here a behavioral mechanism that satisfies these two constraints. Individuals simply modulate their probability to switch to another resource as a function of the local density of conspecifics locally detected. As a result, the individuals gather at the smallest resource that can host the whole group, hence reducing competition and exploitation costs while fulfilling the overall group's needs. Our analysis reveals that the group becomes better at discriminating between similar resources as it grows in size. Also, the discrimination mechanism is flexible and the group readily switches to a better suited resource as it appears in the environment. The collective decision emerges through the self-organization of individuals, that is, in absence of any centralized control. It also requires a minimal individual cognitive investment, making the proposed mechanism likely to occur in other social species and suitable for the development of distributed decision making tools.

## Introduction

Survival of animal groups strongly depends on their ability to select resources that can sustain their population. The decision-making process is usually a combination of exploration and information pooling that leads the group to focus its activity on one or a subset of all the available resources. As reviewed in [1], [2], several types of organization exist that can lead group members to reach a consensus. The final decision can be made by only one individual who occupies a dominant position in the group [3], or it can be the result of a cooperation between all or a part of the group members [4].

Self-organized decision-making processes pertain to this last category [2]. In the literature, one mechanism is frequently used to explain consensus decision making in groups[5], [6]: the probability for an individual to select a given option (for instance, a food source) increases non linearly with the number of conspecifics that select the same option. This creates a positive feedback as more and more individuals tend to make the same choice, and eventually leads to a consensus between members of a group, in a fully distributed way. Well-known examples of these decision-making processes are the collective selection of the richest food source by bees [7] or ants [8]. Insects exploiting richer sources tend to recruit more individuals, biasing the group's choice toward the most rewarding option.

However, more is not always better. Large and rich resources are more likely to attract competitors, adding an extra cost for the defence of the resource [9]–[11]. Moreover, individuals may be forced to spread over a larger space to occupy the whole resource, hence impairing intra-group cooperation or reducing the benefits of group living [12], [13]. In all these situations, it is more advantageous for groups to select resources that correspond closely to their needs, and to avoid oversized ones. But this task requires to evaluate the overall needs of the group in addition to the capacity of the available resources. This may be particularly difficult to achieve with a large population, or if individuals have low cognitive abilities [14].

Here we propose and investigate a decentralized mechanism to discriminate between several resources the one that best fits a group's needs. Our approach requires no explicit communication, minimal cognitive and sensing individual capacities, and is based solely on local interactions between neighboring individuals.

Our starting point is a model proposed by Amé *et al.* [15] to explain the collective choice behavior of cockroaches when they select one shelter out of several identical ones. Amé *et al.* 's model is based on the assumption that the rate 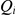 of cockroaches leaving shelter 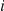 per second decreases with the density 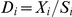 of individuals (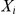) in the shelter (of capacity 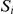):
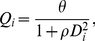
(1)where parameters 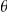 and 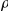 determine the minimum and maximum values of the rate 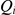 depending on 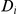. From this rate 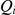, we can directly derive the probability per-unit-time that an individual leaves a shelter. The model predicts that, when each shelter is sufficiently large to house all the cockroaches, the group will aggregate in only one of them. If shelters are too small the model predicts that the group will use two or more shelters equally.

Amé *et al.* restrict their study to the case of identical shelters. Moreover, although they show that the probability per-unit-time for an individual to leave an aggregate is a function of the aggregate's size, they do not indicate how cockroaches could estimate the density of conspecifics in a shelter.

We propose here a broader perspective, in which shelters can be seen as resources for cockroaches and the shelters' surfaces correspond to the capacities of the resources. The total surface of the cockroaches' bodies corresponds to the group needs. Building on this equivalence, we generalize the model of Amé *et al.* to study the behavior of a group when resources of different capacities are available in its environment. These resources can be shelters, as in the case of cockroaches, but more generally they can correspond to any source of supply or support such as food sources, nest site, resting site, etc.

For two resources, the model therefore becomes:
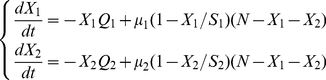
(2)where 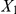 and 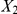 are the average number of individuals at the two resources, 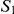 and 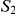 are the capacities of the resources, 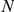 is the total number of individuals, and 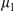 and 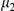 are the probabilities for an individual to encounter each resource during a random walk in a limited space. The factor 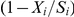 models the saturation of the resources when their maximum capacity is reached. The equations are composed of a positive term that reflects the average number of new individuals using the resource and a negative term reflecting the average number of individuals that leave the resource.

**Figure 1:**
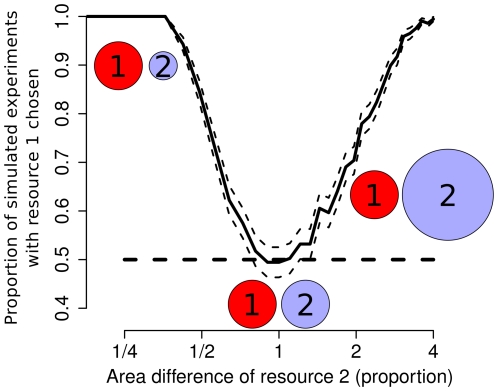
Simulations of the extended model. Resource 1 has a carrying capacity matching the group size while the radius of resource 2 varies (represented with a log-scale). The figure reports the proportion of simulations (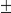 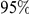 CI) that end with the choice of resource 1 (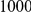 trials). We consider as chosen the resource occupied by the largest number of individuals. When the two resources have a sufficiently different size, the group decisively selects resource 1. When resources have similar size, the model predicts the random choice of one of the resources. Simulations are produced with a discrete event model based on the system of equations 2 in which individuals probabilistically move between the environment and the resources.

Interestingly, the generalized model predicts that the group selects the smallest resource available that is large enough to sustain the group (see Fig. 1), therefore avoiding both undersized and oversized resources. This collective behavior follows from a simple modulation of the individuals' probability per-unit-time to leave as a function of the density of individuals at the resource.

To validate this theoretical prediction, we set up real world and simulated robotics experiments (see *Materials and Methods* for full details). Unlike abstract models based on equations, realistic simulations require very complete specifications of the individuals and their behavior. These simulations allow us to study the collective behavior in a wide range of conditions, varying group size and resource sizes. Physical robots provide a validation of our simulation results and demonstrate the feasibility of the collective behavior in real world systems.

**Figure 2:**
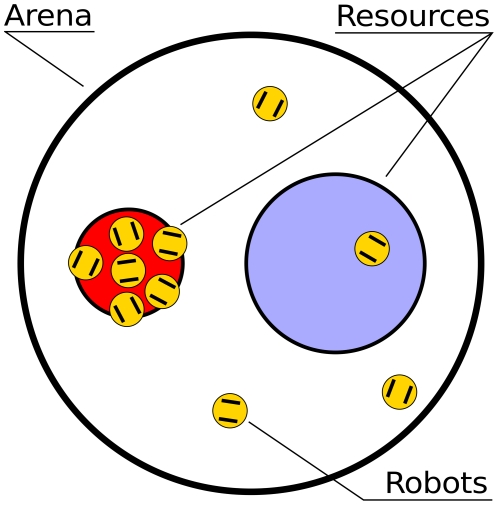
The experimental setup. In a circular arena of 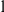 m radius, the two resources are represented by cardstock discs (radius of 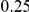 m and 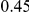 m) fixed to the ground.

**Figure 3:**
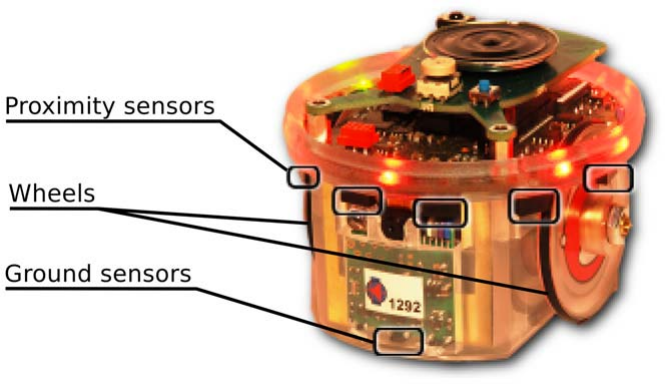
The *e-puck* robot used in our experiments. It has a cylindrical body and moves with two motorized wheels. Perception of obstacles or other robots is achieved through infrared sensors distributed around the body. A sensor directed toward the ground allows perception of resources.

We placed a group of 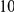 e-puck robots [16] in a circular arena (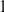 m radius), searching for resources during one hour (see Fig. 2 and 3). The role of resources was played by two cardstock discs over which robots could move freely. Using infrared sensors directed to the ground, robots could detect when they were at a resource. In addition, 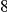 infrared sensors disposed around the body of the robots allowed them to detect obstacles such as arena borders or other robots in a range of 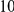 cm or less. The target resource, having a capacity matching the group size, could host all the robots involved in the task. Its dimensions were obtained using simulations (radius of 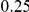 m). The other resource was either larger or smaller. Following the predictions of our mathematical model, we expected the robots to gather on the target resource.

As in the model, robots decide to stay at or leave a resource as a function of the density of robots already present. However, computing the density of a region is a non-trivial task for individuals that have limited perception and cognitive abilities because it requires knowledge of both the surface of the region and the number of conspecifics present in the region. To estimate the region's surface, individuals might localize themselves in their environment and build an internal representation of the region [17], [18]. Another possibility would be to rely on Buffon's needle method, which involves marking of the environment [19]–[21]. Moreover, to count the number of conspecifics in the region, robots should keep track of their encounters and avoid double-counting.

Here, robots have very limited capabilities. They can only perceive whether or not they are on a resource, and locally detect obstacles or other robots. They are not endowed with sufficient perceptual or cognitive abilities to measure the size of the resources nor to count the total number of robots.

To solve the problem of density estimation, we use therefore a method that takes inspiration from a recent study of emigrating ants *Temnothorax albipennis* [22]. These ants rely on the rate of encounters with other ants to evaluate the density of individuals in a cavity: the more contacts they have with other ants, the greater their estimated value of the density. Using this simple mechanism, it is possible to implement the collective selection process described previously.

The behavior of the robots is therefore a combination of the cockroaches' and the ants' behavior [15], [22]. The robots' controllers are implemented as probabilistic finite state machines [23]. When a robot is not at a resource, it performs a random walk with obstacle avoidance till a resource is found again. When at a resource, the robot also performs a random walk, trying to remain there by turning around upon encountering borders. Every 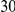 s, the robot can decide with the probability per-unit-time derived from 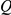 to leave the resource. The density 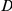 is estimated by the number of collisions with others robots measured during this time interval. The parameters 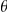 and 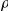 are obtained using a genetic algorithm designed to favor a fast and stable collective choice of the target resource (see *Materials and Methods*).

## Results

### Collective discrimination

**Figure 4:**
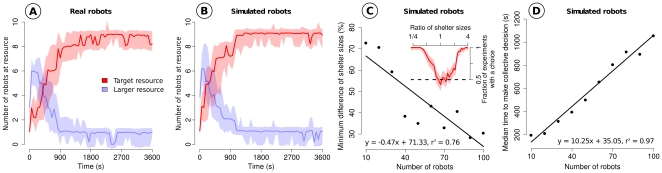
Collective discrimination between two different resources. (**A** and **B**) We use a target resource of ideal dimensions and a larger resource. The figures show the number of robots (median 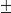 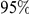 CI) at each resource as a function of time in reality and in simulations respectively (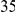 trials). Initially robots find the larger resource more easily, and then their collective choice changes quickly in favor of the target resource. (**C**) Discriminatory power of the collective behavior as a function of the group size. The inset shows the choice of the target resource by a group of 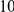 simulated robots when the other resource has different sizes. When the two resources have similar dimensions, the robots are not able to discriminate them properly, and we observe a random choice of the resource (binomial test, 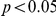). The main plot shows the minimum resource size difference necessary to observe discrimination. This minimum difference decreases with the group size (binomial test with 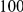 trials, 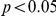). A linear regression performed on the data indicates a significant improvement of the discriminatory power (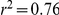, t-test 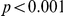). (**D**) Median time to make the collective decision in function of the group size. We report the first time when the target resource contains most robots. The time grows linearly with the number of robots involved in the task (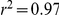, t-test 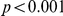).

In a first set of experiments, we assess the robots' capability to discriminate between two different resources. The robots are offered a target resource that provides enough space for the group, while the area of the other one is 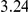 times larger. The average number of robots found at each resource is reported in Fig. 4A. The experiments start with robots randomly scattered in the environment and last one hour. At the end of all the trials, robots have collectively selected the target resource (in the following, we consider as chosen the resource occupied by the largest number of individuals). In the first moments, robots could be found at the large resource because it is most likely to be discovered first. However, the low density of robots at this resource prevented them from remaining there. On the contrary, the target resource, once discovered by the robots, provided them higher densities and therefore longer staying times. Finally, robots were able to discriminate between resources of different sizes, choosing the one that best fits the group size.

### Accuracy and scalability

The second set of experiments sheds light on the discriminatory power of the group. In order to allow a large number of replications, we rely here on simulations that were validated against the first set of experiments (see Fig. 4B, and Videos S1, S2). We first introduce a target resource in the environment. In successive tests, we add resources of growing size and observe which one is chosen by the simulated robots. The size of the presented resources varies from 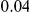 to 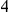 times the area of the target resource. With 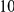 robots, we observe that the group successfully recognizes the target resource when the other resource is smaller or larger by a factor of 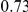 or 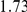 (see inset of Fig. 4C). When the resources do not differ enough, the robots are not able to discriminate them anymore. They are instead making random choices. We measure the difference in resource size needed to observe selection of the target resource for a growing number of robots. To allow comparison of results, we scale the environment size and the duration of the trials with respect to the number of robots used (see *Materials and Methods*). As the number of robots grows, we see a rapid increase in the discriminatory power (see Fig. 4C). With 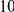 robots, a minimum difference of 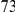
% is necessary to observe selection of the target resource, while 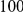 robots only require a difference of 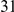
%: larger groups of robots discriminate between resources more accurately. We also report the median time the robots need for making their collective decision in Fig. 4D. The decision time grows linearly with the number of robots involved in the task (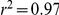).

### Adaptivity

**Figure 5:**
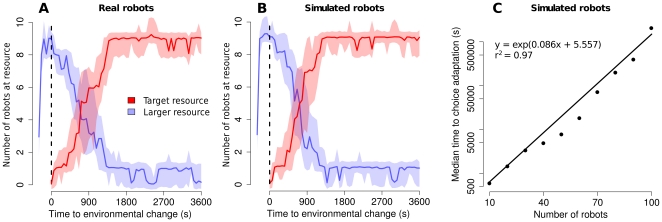
Adaptivity of the collective choice. (**A** and **B**) Ten robots are presented a single large resource and gather in it. After five minutes (dashed line) a target resource is introduced in the environment. Figures show the number of robots (median 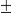 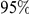 CI) at each resource as a function of time in reality and in simulations respectively (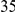 trials). We observe a quick adaptation of the collective choice towards the target resource after its introduction. (**C**) The median adaptation time (plotted in log scale) grows exponentially with the number of robots involved (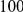 trials). We considered that the choice was reverted as soon as there were more robots at the target resource than at the large resource.

The third set of experiments shows the adaptivity of the robots' collective choice when a better opportunity appears in the environment. We first perform experiments with 10 robots and then explore the impact of increasing the group size with simulations. Experiments start with a single resource in the environment, which is 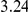 times bigger than the target resource. As this is the only option available, robots aggregate at this resource (see Fig. 5A and B). After five minutes, we add a target resource inside the arena. With 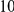 robots, the group adapts its choice to the new settings and selects the target resource. It takes on average 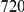 s to observe this adaptation, which we continue to observe in simulation with larger groups of robots. This is shown in Fig. 5C, where we report the median time of adaptation with respect to the number of simulated robots. With 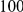 robots, adaptation occurs after 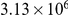 s. Adaptation time grows exponentially with the number of robots involved (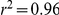).

## Discussion

Our results illustrate how simple interactions can lead a group to collectively choose amongst resources one that closely matches its needs. The collective discrimination arises from the interplay of several factors. On the one hand, individuals prefer to stay at resources where their density is higher. This positive feedback strongly favors the selection of smaller resources where higher densities can be achieved. On the other hand, it is more difficult to join a resource where the density is high. This negative feedback favors the selection of larger resources that can host additional individuals. Moreover, smaller resources are less likely to be discovered and if one is selected the group may be forced to split [15]. Because of this, competition may happen with any other resource found by these excluded individuals. In our model, these factors balance each other out in one case: when the capacity of a resource matches the size of the group.

This collective behavior can be achieved by agents with very limited perceptual and cognitive abilities, as demonstrated by our robotics implementation. In our experiments, robots can only detect when they are at a resource site, and they are neither able to measure the capacities of the different resources nor to evaluate the number of robots using them. Moreover, the robots do not communicate any information explicitly, and solely rely on the detection of nearby robots to make decisions. As a consequence, the collective discrimination process does not require to centralize information, nor to refer to a leader.

We found that the accuracy of the discrimination increases with the group size: larger groups are able to detect proportionally smaller differences between two resources. Similar results have been observed in various biological systems. Groups of *Gasterosteus aculeatus* fish for instance, when their size increases, become better at discriminating phenotypic differences in pictures of conspecifics [24] or at selecting a route where risks of predation are reduced [25]. In *Temnothorax albipennis* ants, when environmental constraints limit the number of scouts able to visit potential nests before colony emigration, the probability of selecting the best available site decreases [26], [27]. In our model, as well as in these biological examples, the better accuracy with increasing group sizes can be explained by the “many wrongs” principle [13], [28], an application of the more fundamental statistical phenomenon known as the law of large numbers. It states that, in any system able to pool individual estimations of a quantity (here the density at a given resource), the confidence interval of the mean value of this quantity (*i.e.*, the accuracy of the estimation) decreases with the number of individuals.

We also observed that the collective choice is flexible. If the group has selected a resource, it is able to switch for a better one introduced afterwards. The ability to adapt in changing environments is triggered by the initial low density of individuals at the primary resource. This favors the exploratory behavior of individuals that are thus able to find the better resource after its introduction.

It is worth noting that the spatial component of our model, and in particular the exploratory behavior of robots, has a major impact on the dynamics of the discrimination process. We found for instance that the time to make a decision grows linearly with the number of robots involved in the task. But we also showed that the adaptation time increases exponentially with the group size. In our simulations, the setup is scaled with respect to the target resource corresponding to the group size but the speed of the robots is kept constant. Therefore, the time for individuals to switch between resources grows linearly with the size of the environment and the group size, and so does the time to make a decision. Similarly, the random walk of the robots is not modified when the resources and the environment were enlarged. In adaptation experiments with large groups of robots, this increased the probability that robots, initially grouped at a less suitable resource, came back toward it, thus reducing the probability to find the more suitable resource.

The simplicity of our theoretical model and of its robotics implementation suggests that a similar mechanism could exist in nature. For instance, this mechanism may explain the pattern of shelter selection in the den-dwelling Caribbean spiny lobster. Indeed, individuals of this species tend to aggregate under shelters maximizing their density when predation risks are high, and they select shelters that are scaled to their group size [29]. In the ant *Temnothorax albipennis*, nests are scaled according to the size of the colony [30] and ants select new nest sites that match their colony size [31]. Although collective decisions and resource selection processes have been long studied in social insect species, such as ants [8, 32 33], bees [7], [34] and cockroaches [35], the effects of density at the resource have been neglected. In all these studies, animals are presented with several feeders containing various food quantities, often in the form of a sucrose solution. However, feeders in these experiments are always of identical and small size, thus masking possible effects of density at the resources. Though, density of conspecifics is information that could be used by social animals to evaluate to which extent a resource is exploited [36] or has been secured against competitors. The collective discrimination mechanism we introduced here, relying on local estimates of the density at a resource, could help them to achieve a compromise between the benefits associated with small and large resources and the costs of their exploitation. To assess this question, it would be interesting to reproduce classical experiments of food source selection by using food patches of different dimensions, or to evaluate during field observations the relationship between the size of a group and the size of the resources it exploits.

Recent studies have highlighted the mechanisms that animal societies use to solve complex problems through simple and highly distributed interactions [5], [6], [12], [13], [37]. They have attracted a lot of attention from the computer science, the operational research and the robotics communities because their distributed nature gives them several advantages over centralized control algorithms [38], [39]. They are often stated as more robust (several copies of each component exist), scalable (no communication bottleneck) and cost effective (identical components are easier to mass produce). In this context, we believe that our results open interesting perspectives for the development of distributed resource management systems, especially when group's needs and/or resource availabilities are dynamical and difficult to evaluate.

## Materials and Methods

### Experimental setup

The environment in which experiments take place is a circular arena of 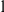 m radius (see Fig. 2). Since robots' perception relies on measures of infrared light, the arena is enclosed in a room without window to prevent natural light from entering the setup. Two compact 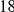 watt fluorescent lamps placed 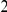 m above the arena shed light in the room. The role of resources is played by two dark cardstock discs fixed to the ground. One resource, called the target resource, has a carrying capacity that matches the number of robots (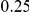 m radius). The other resource is larger than the target by a factor of 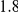 (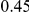 m radius). We recorded the experiments with a camera placed above the setup. Data was extracted from the videos using a tracking system designed at the IRIDIA laboratory that identified how many robots were at each resource.

### Robotic platform

We use *e-puck* robots (see Fig. 3) designed by Francesco Mondada and Michael Bonani at the École Polytechnique Fédérale de Lausanne (EPFL), Switzerland [16]. E-pucks are modular, robust and inexpensive robots designed for research and educational purposes. The robots have a cylindrical body (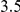 cm radius) and move using a differential drive system made of two wheels directly fixed to the shafts of stepper motors. Perception of the environment is achieved using infrared sensors. Robots perceive obstacles and other robots by periodically sending 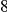 infrared beams in opposite directions. The intensity of reflected infrared light informs the robots about nearby objects. Moreover, robots perceive resources using an infrared sensor directed to the ground. Additional information and free software regarding the *e-puck* robot are available at www.e-puck.org.

### Simulator

Simulation results were obtained using the *Twodeepuck* simulator, a fast multi-robot simulator coded in C++ initially designed by Anders L. Christensen and Laurent Bury at the IRIDIA laboratory [40], [41]. Motion of the robots is simulated with standard two dimensional kinematics as described in [42]. In order to accurately reproduce real world experiments, we have systematically sampled the data output of the robot's infrared sensors. We gathered the signal intensity perceived when the robot was presented another robot or a wall. To get a complete picture of the sensor's output, we tested an exhaustive set of distances and angles. With this data at hand, we created models of the sensors output. The data fed to the controllers in simulation corresponds closely to what happens in reality.

### Robotic controller

Robots are controlled by a finite state machine. In the following we summarize the possible behavioral states. The controller is initialized in the *Explore* state.

- **Explore.** The robot performs a random walk in the environment. An obstacle avoidance subroutine is triggered when needed. The robot switches to the *Stay* state when it encounters a resource.
- **Stay.** The robot performs a random walk inside the resource. Every 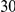 seconds, the robot decides with probability per-unit-time 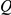 to leave the resource and enter the *Explore* state. If the robot finds itself outside the resource it switches to the *BackToResource* state.
- **BackToResource.** The robot performs a U-turn then keeps turning on the spot until it detects the resource again. If the robot finds itself in the resource it switches to the *Stay* state. If 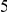 seconds have elapsed and the robot still does not perceive the resource, it switches to the *Explore* state.

### Parameters tuning

The parameters 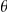 and 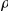 determine when robots make the decision to leave the resources. For a robot, the probability per-unit-time 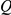 to leave a resource is expressed as 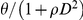, where 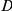 is an estimate of the robot density in the neighborhood. If the resource is crowded, 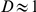, and 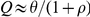. If the resource is empty, 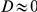, and 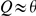. Therefore the parameters 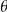 and 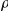 determine the maximum and minimum rate of robots leaving a resource. From this rate, we directly derive (they are equal) the probability per-unit-time 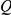 of a single robot to leave a resource.

To ensure an effective collective behavior (robots' batteries get discharged in less than 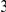 hours), we tune these parameters with a simple generational genetic algorithm [43]. We define a genotype as a vector of two real values to be assigned to 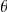 and 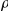. We run the genetic algorithm for 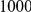 iterations, during which we breed new generations of 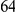 genotypes. The genetic algorithm loop consists in the evaluation, the selection and the reproduction of the genotypes.

To evaluate the fitness of a given genotype, we parameterize the controller of 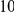 simulated robots with the genotype. We run 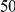 simulated experiments with a target resource and a larger resource (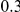 m radius). We also run 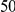 experiments with a target resource and a smaller resource (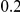 m radius). The fitness of the evaluated genotype is computed as an indicator of the ability of the robots to make a choice that is fast, lasting, and in favor of the target resource:
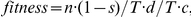
where 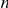 is the proportion of experiments in which a collective choice of the robots occured, 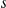 is the average starting time of the choices, 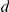 is the average duration of the choices, 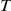 is the total duration of an experiment and 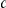 is the proportion of choices made in favor of the target resource.

After evaluation, we rank the genotypes according to their fitness and create a new generation. The best 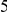
% genotypes are cloned. Then, genotypes are picked randomly from the best 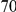
% and mutated with a probability of 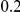 or reinitialized randomly with a probability of 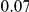. A mutation consists of adding to the genotype random values drawn from Gaussian distributions. For 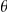, we use a Gaussian with 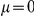 and 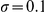. For 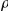, we use a Gaussian with 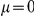 and 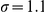. During evolution, all vector component values are constrained to remain within the ranges 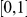 for 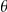, and 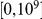 for 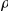.

The analysis of the results reveals that the collective behavior of a group of 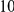 robots in our experiments is the most effective when 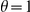 and 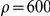.

### Scaling the setup when group size increases

Simulated experiments involve up to 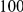 robots. Because larger groups of robots span over a larger surface and need more resources, we have to scale the size of the arena and the size of the resources with respect to the group size considered. Also, since the robots do not move faster and the arena is enlarged, we have to scale the duration of the experiments. In order to make meaningful comparisons of the results accross different group sizes, we ensure that for any size of the target resource a single robot alone in the setup spends the same proportion of the experiment duration looking for the resources. To this end, we keep a constant ratio between the size of the target resource and the size of the arena [44], and that same ratio is also used to scale the duration of the experiments. Therefore, the whole scaling procedure depends solely on the size of the target resource. The size of the target resource is identified using simulations in which resources of various sizes are presented to the robots. The target resource is the one constantly preferred by the group. For each group size considered, Table 1 summarizes the target resource size, arena size and experiments duration we used to parameterize our experiments.

**Table 1:** Parameters' values.

Group size	Target resource radius (m)	Arena radius (m)	Experiment duration (s)
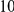	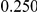	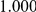	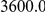
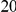	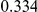	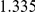	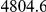
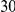	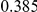	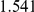	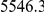
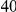	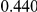	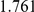	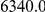
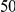	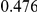	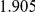	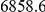
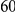	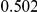	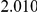	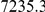
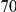	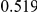	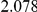	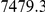
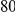	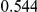	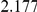	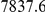
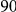	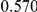	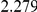	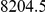
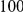	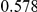	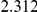	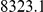

Summary of the main parameters' values used in our experiments with respect to the group size considered.

## Supporting Information

Video S1 **Collective discrimination with a large resource and a target resource.** The video shows a simulated experiment in which 10 robots are randomly placed in an environment with a target resource and a larger resource. After one hour, the group of robots has selected the target resource.
(MP4)

Video S2 **Collective discrimination with a small resource and a target resource.** The video shows a simulated experiment in which 10 robots are randomly placed in an environment with a target resource and a smaller resource. After one hour, the group of robots has selected the target resource.
(MP4)

## References

[1] Conradt L, List C (2009). Group decisions in humans and animals: a survey.. Philosophical Transactions of the Royal Society of London Series B, Biological sciences.

[2] Conradt L, Roper TJ (2005). Consensus decision making in animals.. Trends in Ecology & Evolution.

[3] King AJ, Cowlishaw G (2009). Leaders, followers and group decision-making.. Communicative & Integrative Biology.

[4] Sumpter DJT, Pratt SC (2009). Quorum responses and consensus decision making.. Philosophical Transactions of the Royal Society of London Series B, Biological sciences.

[5] Camazine S (2001). Self-organization in biological systems..

[6] Garnier S, Gautrais J, Theraulaz G (2007). The biological principles of swarm intelligence.. Swarm Intelligence.

[7] Seeley T, Camazine S, Sneyd J (1991). Collective decision-making in honey bees: how colonies choose among nectar sources.. Behavioral Ecology and Sociobiology.

[8] Beckers R, Deneubourg JL, Goss S, Pasteels JM (1990). Collective decision making through food recruitment.. Insectes Sociaux.

[9] Nagamitsu T, Inoue T (1997). Aggressive foraging of social bees as a mechanism of oral resource partitioning in an Asian tropical rainforest.. Oecologia.

[10] Holway DA, Case TJ (2001). Effects of colony-level variation on competitive ability in the invasive Argentine ant.. Animal Behaviour.

[11] Lichtenberg EM, Imperatriz-Fonseca VL, Nieh JC (2010). Behavioral suites mediate group-level foraging dynamics in communities of tropical stingless bees.. Insectes sociaux.

[12] Krause J, Ruxton GD (2002). Living in groups..

[13] Sumpter DJT (2010). Collective Animal Behavior..

[14] Seeley TD (2002). When is self-organization used in biological systems?. Biological Bulletin.

[15] Amé JM, Halloy J, Rivault C, Detrain C, Deneubourg JL (2006). Collegial decision making based on social amplification leads to optimal group formation.. Proceedings of the National Academy of Sciences of the United States of America.

[16] Mondada F, Bonani M, Raemy X, Pugh J, Cianci CM (2009). The e-puck, a robot designed for education in engineering.. Proceedings of the 9^th^ Conference on Autonomous Robot Systems and Competitions.

[17] Durrant-Whyte H, Bailey T (2006). Simultaneous localization and mapping: part I. IEEE Magazine on Robotics & Automation.

[18] Hafner VV (2005). Cognitive maps in rats and robots.. Adaptive Behavior.

[19] Şahin E, Girgin S, Uğur E (2006). Area measurement of large closed regions with a mobile robot.. Autonomous Robots.

[20] Girgin S, Sahin E (2004). Blind area measurement with mobile robots.. Proceedings the 8^th^ Conference on Intelligent Autonomous Systems.

[21] Franks N, Mallon E, Bray H, Hamilton M, Mischler T (2003). Strategies for choosing between alternatives with different attributes: exemplified by house-hunting ants.. Animal behaviour.

[22] Pratt SC (2005). Quorum sensing by encounter rates in the ant Temnothorax albipennis.. Behavioral Ecology.

[23] Rabin MO (1963). Probabilistic automata.. Information and Control.

[24] Sumpter DJT, Krause J, James R, Couzin ID, Ward AJW (2008). Consensus decision making by fish.. Current Biology.

[25] Ward AJW, Sumpter DJT, Couzin ID, Hart PJB, Krause J (2008). Quorum decision-making facilitates information transfer in fish shoals.. Proceedings of the National Academy of Sciences of the United States of America.

[26] Pratt SC, Sumpter DJT (2006). A tunable algorithm for collective decision-making.. Proceedings of the National Academy of Sciences of the United States of America.

[27] Marshall JAR, Dornhaus A, Franks NR, Kovacs T (2006). Noise, cost and speed-accuracy trade-offs: decision-making in a decentralized system.. Journal of the Royal Society Interface.

[28] Simons AM (2004). Many wrongs: the advantage of group navigation.. Trends in Ecology & Evolution.

[29] Eggleston DB, Lipcius RN (1992). Shelter selection by spiny lobster under variable predation risk, social conditions, and shelter size.. Ecology.

[30] Franks NR, Wilby A, Silverman BW, Tofts C (1992). Self-organizing nest construction in ants -sophisticated building by blind bulldozing.. Animal Behaviour.

[31] Mallon EB, Franks NR (2000). Ants estimate area using Buffon's needle.. Proceedings of the Royal Society of London Series B, Biological Sciences.

[32] Beckers R, Deneubourg JL, Goss S (1993). Modulation of trail laying in the ant Lasius niger (Hymenoptera: Formicidae) and its role in the collective selection of a food source.. Journal of Insect Behavior.

[33] Pasteels JM, Deneubourg JL, Goss S (1987). Self-organization mechanisms in ant societies (I): trail recruitment to newly discovered food sources.. From individual to collective behavior in social insects.

[34] Seeley TD (1995). The wisdom of the hive: the social physiology of honey bee..

[35] Lihoreau M, Deneubourg JL, Rivault C (2010). Collective foraging decision in a gregarious insect.. Behavioral Ecology and Sociobiology.

[36] Mailleux AC, Deneubourg JL, Detrain C (2000). How do ants assess food volume?. Animal Behaviour.

[37] Couzin ID (2009). Collective cognition in animal groups.. Trends in Cognitive Sciences.

[38] Eberhart RC, Shi Y, Kennedy J (2001). Swarm Intelligence..

[39] Bonabeau E, Dorigo M, Theraulaz G (1999). Swarm intelligence: from natural to artificial systems..

[40] Christensen AL (2005). Effcient neuro-evolution of hole-avoidance and phototaxis for a swarm-bot..

[41] Bury L (2007). Conception et implémentation en c++ d'un simulateur pour les robots e-puck et réalisation de tests de validation pour la cinématique de base..

[42] Dudek G, Jenkin M (2010). Computational principles of mobile robotics..

[43] Goldberg DE (1989). Genetic algorithms in search, optimization and machine learning..

[44] Blanco S, Fournier R (2003). An invariance property of diffusive random walks.. Europhysics Letters.

